# Supplementary material for: COVID-19 and social media: Beyond polarization
Source: PNAS Nexus. 2023 Aug 1;2(8):pgad246. doi: 10.1093/pnasnexus/pgad246 (PMC10411931; doi:10.1093/pnasnexus/pgad246)
Supplement: pgad246_Supplementary_Data [file pgad246_supplementary_data.pdf]

Supplementary Material

COVID-19 and social media: Beyond polarization

Giacomo De Nicola[a,*], Victor H. Tuekam Mambou[b,a], Göran Kauermann[a]

Department of Statistics, LMU Munich[a]
ifo Institute – Leibniz Institute for Economic Research at the University of Munich, Germany[b]

The following contains technical details and supplementary information to the manuscript. The full data and code to reproduce the analysis and to aid the reader in applying the presented models to their own data can be found in our Github repository, available at https://github.com/gdenicola/latent-space-covid-twitter-elites.

S1 Defining the set COVID-19 Twitter elites

Social media elites can be broadly understood as "users with the ability to influence" [1]. The concept is further widely discussed in the literature [2–4]. While there is no unique, precise definition of it, the idea can further be narrowed down: The term "social media elite" typically refers to a group of highly influential and popular users with extensive reach on the platform and who have a significant impact on conversations, trends, and narratives circulating online. These users often include celebrities, politicians, journalists, thought leaders, and influencers who have a large and engaged audience and are frequently retweeted, quoted, and mentioned by others. This characterization, while informative, is quite broad, and by itself does not indicate a unique way of identifying elites in practice. Operational definitions for empirical applications are often based on engagement metrics, such as the number of followers of each user, as well as tweet likes, retweets, quotes, and replies. Choices within these metrics are virtually endless and depend on both the data at hand and the research question that is tackled. In our case, the focus lies on analyzing the behavior of users who actively participate in the Twitter conversation on COVID-19, and that have the ability to influence it, i.e. reach many other users. To do so, we made use of a dataset containing a 1% sample of all tweets containing keywords relating to the pandemic in 2021. Based on that, we opted to identify COVID-19 Twitter elites as accounts who authored popular tweets on the topic, i.e. with a total popularity score (given by the sum of likes, retweets, and replies) over a certain threshold, which in our case was set to 2000. Different solutions, such as using the total number of followers, or the total popularity score across all tweets, would also be viable, and were considered. We eventually ended up with our criterion for several reasons:

(a) Firstly, using the total number of followers would tend to include general celebrities, such as e.g. famous actors or pop stars, who only sporadically tweet about the pandemic, possibly with little engagement. Conversely, this criterion would tend to exclude people who are not generally famous, but play an active role in the COVID conversation, and are thus able to reach many users with some of their tweets. While, of course, tweet engagement for a certain user is highly correlated with their number of followers, our focus lies more on the COVID-related, tweet-specific engagement. We, therefore, opted for using direct measures of tweet engagement instead of following counts in our operational definition.

(b) Secondly, we decided against using the total popularity of all tweets by a given user over the analyzed period to identify elites because we are interested in accounts that can produce tweets reaching many people. In contrast, using total engagement across all tweets (without any cutoff for the popularity of single tweets) would include accounts that may garner many total shares because they produce an abundant amount of low-quality posts that each happen to get a little amplification. Examples of these types of accounts may be bots or spam accounts, as discussed by Gallagher et al. [4]. We, therefore, only include users who authored tweets that can be considered impactful on their own. Nonetheless, we thought it would be useful to also carry out a secondary analysis using this alternative criterion based on total popularity, as an additional robustness check for our results. The results of the latter analysis are included in Section S2, and show that our core results are robust to a criterion shift in this direction.

We finally note that a way to further strengthen our criterion towards "sustained" as opposed to "episodic" influence would be to require users to have authored at least two popular tweets included in the sample. This definition lowers the risk of single users ending up in the network by pure chance, i.e. by only having produced very few popular tweets without being a "true" elite. This, of course, comes at the cost of randomly excluding some true elites who may have been only selected in our 1% sample a single time despite having many popular tweets. Given that a 1% sample is already likely to exclude important users, we decided that it was enough to have a single popular tweet to be included. Nonetheless, we additionally ran the analysis using this stricter criterion for comparison purposes, and included the results in the following section.

*Corresponding author: giacomo.denicola@stat.uni-muenchen.de

S2 Robustness checks

In this section, we repeat the analysis performed in the main body of the paper using different criteria for users to be included in the network of COVID-19 Twitter elites. This is meant to showcase the robustness of our results to variations in the choice of popularity threshold as well as structural changes in the inclusion criterion itself. As a reminder, in the main analysis a user was included in the elite network if they authored at least one tweet with popularity score ≥ 2000, where the popularity score is given by the sum of likes, replies and retweets (including mentions). We here performed four additional analyses, each with a different inclusion criterion. These include:

(a) Increasing the popularity threshold from 2000 to 4000, i.e. a user is included in the network if they authored at least one tweet with a total popularity score of 4000;

(b) Lowering the popularity threshold from 2000 to 1000, i.e. a user is included in the network if they authored at least one tweet with a total popularity score of 1000;

(c) Strengthening the definition of elite by requiring at least two popular tweets (i.e. with popularity ≥ 2000) to be included in the network (instead of at least one tweet);

(d) Changing the definition of elite by allowing users who obtained a total popularity score ≥ 5000 across all tweets (instead of in a single one) into the network.

All these different inclusion criterias result in either smaller or bigger elite sets by allowing more or less users into the network. After obtaining the networks, we fit the latent cluster random effects model for each of them, and plotted the results in Figure S1 to provide a visual comparison of the positions of the different nodes. From the figure we can appreciate how the size of the network varies significantly with the criterion, but also that, while there is some minor nodal movement, the positions of the labeled actors are quite similar across the different networks. Even in Figure S1d, i.e. the one depicting the network with total popularity instead of single-tweet popularity as its inclusion criterion, the positioning of the labeled nodes does not seem to exhibit major differences with respect to the original analysis.

To go beyond visual comparison, in addition to the plots we also constructed four confusion matrices. The matrices, shown in Figure S2, compare the classification of users included in the main analysis with those obtained with each of the alternative elite networks. Note that not all users originally included in the elite network are present in the alternative networks due to the varying criterion and the resulting network size, but we can still compare the classification of users included in the main elite network which are still included in the alternative one. As an example, the first row of the matrix in Figure S2a can be interpreted as follows: 12.4% of users were classified in the orange group in both the main analysis and the alternative one, while 0% were classified as blue by the main analysis and as orange in the alternative one. Further, 1.5% of users were classified as orange in the main and as blue in the alternative, while 86.1% were classified as blue by both analyses. Looking at the four matrices, we can see that the vast majority of users that are present in both networks are classified in the same way, with only a few users switching communities with respect to the original labeling. Overall, these results thus demonstrate a good degree of robustness of our results to changes in the inclusion criterion, or, in other words, that the results are not very sensitive to the choice of elite definition being used.

S3 Word frequency

In the main body of the paper, we stated that vaccination was a prominent theme in the COVID-related discussion in 2021. To corroborate this, in Figure S3 we provide a bar chart depicting the frequency of the top 15 content words across the complete sample. English translations of the words are included in parentheses where relevant. The table shows that, after "covid19", "coronavirus", "corona", and "mehr" (which means "more"), the word "impfung" (meaning "vaccine") appears most often. Given that the word was not used to filter the dataset (see [5]), this gives an indication that vaccination was a central theme in the COVID-19-related Twitter conversation.

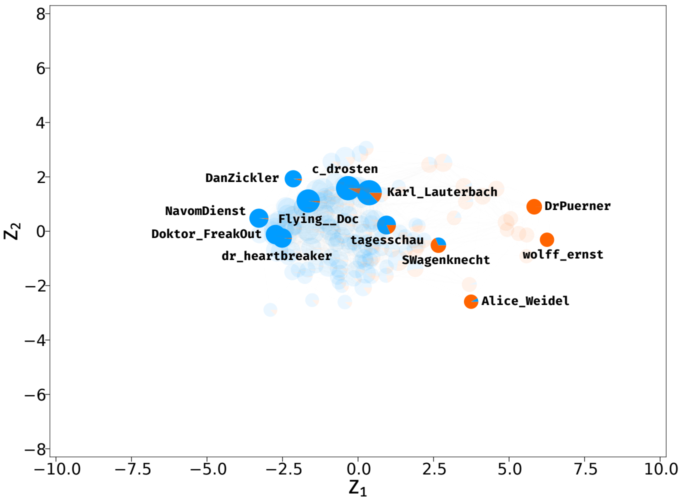

(a) Latent positions of users who authored at least one tweet with popularity score ≥ 4000 (137 users).

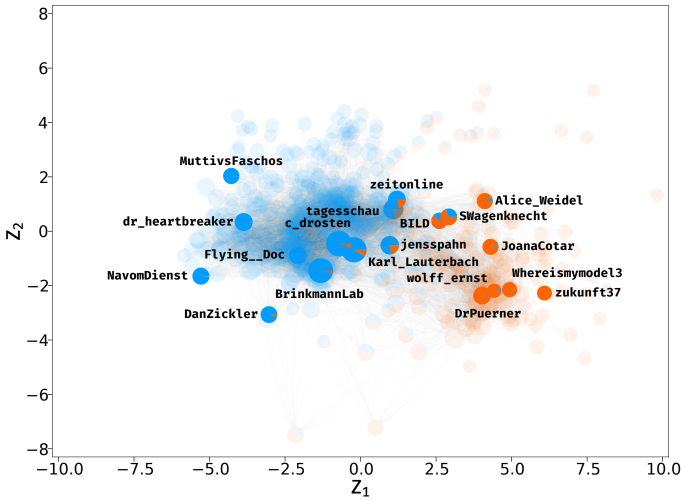

(b) Latent positions of users who authored at least one tweet with popularity score ≥ 1000 (730 users).

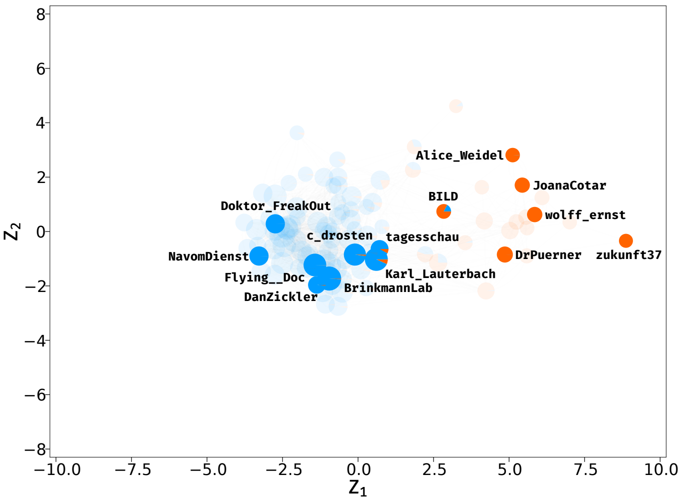

(c) Latent positions of users who authored at least two tweets with popularity score ≥ 2000 (99 users).

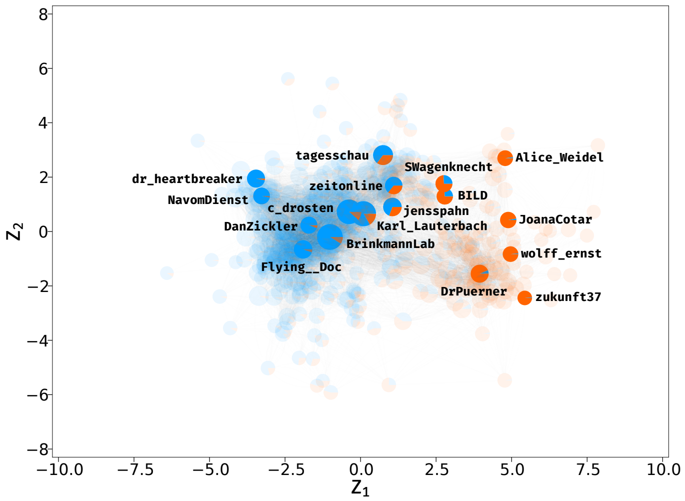

(d) Latent positions of users with a cumulative popularity score across all tweets ≥ 5000 (516 users).

Figure S1: Latent positions of the actors in the network of COVID-19 Twitter elites estimated via the latent cluster random effects model using different popularity criteria. A number of notable users are highlighted.

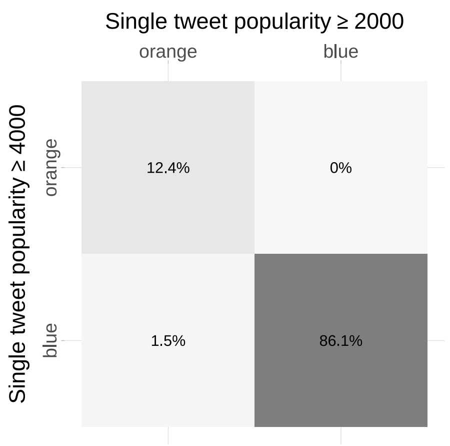

(a) Confusion matrix for classifications in Figure S1a versus main analysis (Figure 3).

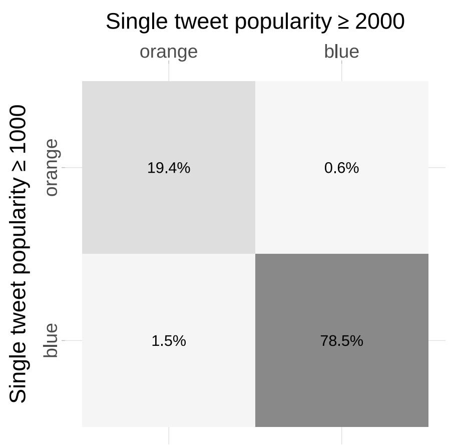

(b) Confusion matrix for classifications in Figure S1b versus main analysis (Figure 3).

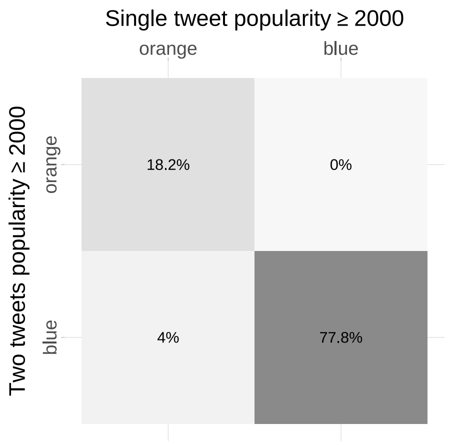

(c) Confusion matrix for classifications in Figure S1c versus main analysis (Figure 3).

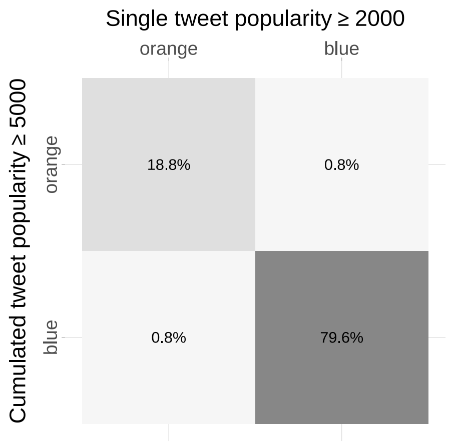

(d) Confusion matrix for classifications in Figure S1d versus main analysis (Figure 3).

Figure S2: Comparison of user classifications obtained via the latent cluster random effects model using different popularity criteria. The confusion matrices included in the figure respectively compare classifications obtained using the networks in Figures S1a, S1b, S1c, S1d against those obtained with the main network (Figure 3).

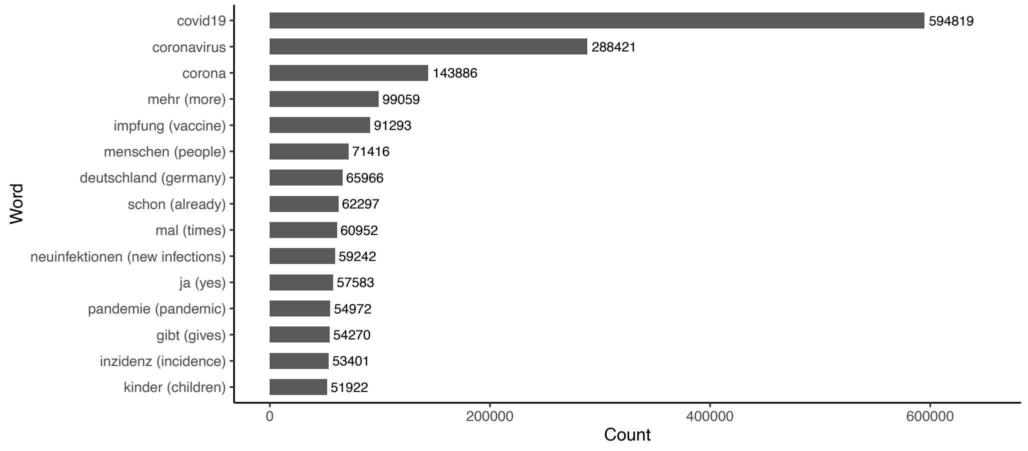

Figure S3: Frequency of the top 15 content words across the whole sample of tweets. The chart shows that vaccination was a central theme in the COVID-19-related Twitter discussion in 2021.

References

[1] Dubois, E. and Gaffney, D. "The multiple facets of influence: Identifying political influentials and opinion leaders on Twitter". *American Behavioral Scientist* 58.10 (2014), 1260–1277.

[2] Papacharissi, Z. and Fatima Oliveira, M. de. "Affective news and networked publics: The rhythms of news storytelling on #Egypt". *Journal of communication* 62.2 (2012), 266–282.

[3] Jackson, S. J. and Foucault Welles, B. "#Ferguson is everywhere: Initiators in emerging counterpublic networks". *Information, Communication & Society* 19.3 (2016), 397–418.

[4] Gallagher, R. J., Doroshenko, L., Shugars, S., Lazer, D., and Foucault Welles, B. "Sustained online amplification of COVID-19 elites in the United States". *Social Media + Society* 7.2 (2021).

[5] Banda, J. M. et al. "A large-scale COVID-19 Twitter chatter dataset for open scientific research—an international collaboration". *Epidemiologia* 2.3 (2021), 315–324.
